# Angiographic complete revascularization versus incomplete revascularization in patients with diabetes mellitus

**DOI:** 10.1186/s12933-022-01488-7

**Published:** 2022-04-19

**Authors:** Doyeon Hwang, Jiesuck Park, Han-Mo Yang, Seokhun Yang, Jeehoon Kang, Jung-Kyu Han, Kyung Woo Park, Hyun-Jae Kang, Bon-Kwon Koo, Hyo-Soo Kim

**Affiliations:** Department of Internal Medicine and Cardiovascular Center, Seoul National University Hospital, 101 Daehak-ro, Jongro-gu, Seoul, 03080 Republic of Korea

**Keywords:** Coronary artery disease, Diabetes mellitus, Complete revascularization, Drug-eluting stent, Outcomes

## Abstract

**Background:**

Considering the nature of diabetes mellitus (DM) in coronary artery disease, it is unclear whether complete revascularization is beneficial or not in patients with DM. We investigated the clinical impact of angiographic complete revascularization in patients with DM.

**Methods:**

A total of 5516 consecutive patients (2003 patients with DM) who underwent coronary stenting with 2nd generation drug-eluting stent were analyzed. Angiographic complete revascularization was defined as a residual SYNTAX (SYNergy between percutaneous coronary intervention with TAXus and cardiac surgery) score of 0. The patient-oriented composite outcome (POCO, including all-cause death, any myocardial infarction, and any revascularization) and target lesion failure (TLF) at three years were analyzed.

**Results:**

Complete revascularization was associated with a reduced risk of POCO in DM population [adjusted hazard ratio (HR) 0.70, 95% confidence interval (CI) 0.52–0.93, p = 0.016], but not in non-DM population (adjusted HR 0.90, 95% CI 0.69–1.17, p = 0.423). The risk of TLF was comparable between the complete and incomplete revascularization groups in both DM (adjusted HR 0.75, 95% CI 0.49–1.16, p = 0.195) and non-DM populations (adjusted HR 1.11, 95% CI 0.75–1.63, p = 0.611). The independent predictors of POCO were incomplete revascularization, multivessel disease, left main disease and low ejection fraction in the DM population, and old age, peripheral vessel disease, and low ejection fraction in the non-DM population.

**Conclusions:**

The clinical benefit of angiographic complete revascularization is more prominent in patients with DM than those without DM after three years of follow-up. Relieving residual disease might be more critical in the DM population than the non-DM population.

*Trial registration* The Grand Drug-Eluting Stent registry NCT03507205.

**Supplementary Information:**

The online version contains supplementary material available at 10.1186/s12933-022-01488-7.

## Introduction

Diabetes mellitus (DM) is one of the major cardiovascular risk factors, which has been considered as a risk-equivalent to coronary artery disease (CAD) [1]. DM population is prone to have a higher prevalence of CAD, which often manifests as multivessel or diffuse disease [2, 3]. DM is also related to vulnerable plaque features, such as high plaque burden or lipid-rich plaque [2]. Patients with DM who underwent percutaneous coronary intervention (PCI) tend to be associated with a worse prognosis than those without DM due to an increased risk of stent restenosis and thrombotic obstruction [4–7]. All of these lead to more challenging PCI in the DM population.

Coronary revascularization by PCI is one of the standard treatment strategies of CAD [8]. Routine invasive therapy has been shown to reduce adverse events in acute coronary syndrome patients [9]. Recent Bayesian analyses demonstrated that coronary revascularization was associated with a lower probability of future cardiac events and relief of angina compared to medical therapy [10, 11]. In clinical practice, completeness of coronary revascularization after PCI is generally of interest as it was reported to be associated with better cardiovascular outcomes [12–16]. Recent studies reported the benefits of complete revascularization in diverse population groups, including ST-segment elevation myocardial infarction [17, 18]. However, the benefit of complete revascularization for CAD in the DM population has not been fully verified in the contemporary drug-eluting stent (DES) era. Considering the nature of DM in CAD, it is unclear whether complete revascularization, relieving all visible lesions by PCI, is beneficial or not in the DM population. Therefore, we sought to evaluate the clinical impact of angiographic complete revascularization among patients with DM who underwent PCI with contemporary DES.

## Methods

### Study population

The study population was from the Grand Drug-Eluting Stent registry (ClinicalTrials.gov Identifier: NCT03507205), a composite of five different multicenter nationwide registries in Korea. The EXCELLENT and RESOLUTE-Korea registry were registries for everolimus-eluting stents (EES; Xience V/Promus) and zotarolimus-eluting Resolute stents (ZES-R; Endeavor Resolute), respectively, and they enrolled all-comers with at least one of EES or ZES-R from 2008 to 2010. The HOST-PRIME and HOST-RESOLINTE registries were dedicated registries for the next generation of EES (Xience Prime, 2010–2012) and ZES-R (Resolute Integrity, 2010–2014), respectively. Lastly, the HOST-BIOLIMUS registry was a registry for the biodegradable polymer-coated biolimus-eluting stents (BD-BES; Biomatrix, Biomatrix Flex, or Nobori) and enrolled all patients with at least one BD-DES from 2011 to 2014. These registries did not have exclusion criteria, except for the patient’s withdrawal. Finally, the Grand Drug-Eluting Stent registry enrolled 17,286 patients from 55 participating centers in Korea (Additional file 1: Table S1). We excluded patients with acute myocardial infarction (MI), and this study was limited to patients with unstable angina or stable CAD. We analyzed patients who underwent PCI using 2nd generation DES and were available with SYNTAX (SYNergy between percutaneous coronary intervention With TAXus and cardiac surgery) score. As the quantitative coronary analysis data was not available in the HOST-BIOLIMUS registry, we excluded this registry from the analysis. We also excluded the patients with a previous history of revascularization. We defined DM patients with a history of DM under medication or fasting blood glucose ≥ 126 mg/dL. All patients were diagnosed with Type 2 DM in our study. The study protocol was approved by the ethics committee at each participating center and followed the principles of the Declaration of Helsinki. All patients provided written informed consent.

### Definition of angiographic complete revascularization

SYNTAX score has been used to identify high-risk CAD patients by evaluating the anatomical complexity of CAD, such as lesion location, severity, and complexity [19, 20]. The residual SYNTAX score was calculated from residual obstructive coronary lesions after PCI, reflecting the residual anatomic disease burden [15]. Previous studies reported the prognostic value of residual SYNTAX score and the usefulness in defining angiographic complete revascularization [15, 18, 21]. We defined angiographic complete revascularization as the residual SYNTAX score of 0. All SYNTAX score was calculated in the core laboratory at Seoul National University Hospital by experienced independent analysts.

### Interventional procedure and medications

PCI was performed based on standard techniques. A loading dose of aspirin (300 mg) and clopidogrel (300–600 mg) was prescribed to all patients before PCI according to their previous medication. After PCI, aspirin (100 mg) was prescribed indefinitely, and P2Y12 inhibitors and other medications, such as beta-blockers, angiotensin-converting enzyme inhibitors, or angiotensin receptor blockers, statins, and calcium-channel blockers, were prescribed according to the physician’s discretion. All procedural decision was also at the physician’s discretion, including the type of stents, coronary imaging usage, procedural techniques, and the use of glycoprotein IIb/IIIa inhibitors.

### Clinical outcomes and follow-up of patients

The primary outcome was the patient-oriented composite outcome (POCO) at 3 years, a composite of all-cause death, any myocardial infarction, and any clinically driven revascularization. The secondary outcome was the stent-oriented composite outcome, target lesion failure (TLF) at three years, including cardiac death, target-vessel related MI, and clinically driven target lesion revascularization (TLR). All deaths were considered of cardiac origin unless definite non-cardiac causes were proven. All clinical outcomes followed the definitions of the Academic Research Consortium [22].

Clinical follow-up was performed at 1, 3, 9, 12, 24, and 36 months by outpatient clinic visit or telephone call. The vital status was cross-checked in all patients by the patient unique identification number of the Korean health care system. The median follow-up duration of all patients was 1125 days with an interquartile range (IQR) of 1093–1140 days.

### Statistical analysis

Categorical variables were summarized by counts and percentages, with comparisons by the Chi-squared test, while continuous variables were summarized by means and standard deviations, with comparisons by the Student’s t-test. The cumulative incidence of each clinical outcome was calculated using Kaplan–Meier estimates at three years, and the Log-rank test was used to estimate the group differences. Cox proportional hazard regression model, stratified by the including centers, was used to estimate the hazard ratio (HR) and 95% confidence interval (CI). Multivariate adjusted HR and its 95% CI was derived from the multivariate Cox proportional hazard regression model, including the following patient risk factors: age, sex, body mass index, hypertension, hypercholesterolemia, heart failure, chronic kidney disease, family history of CAD, previous cerebrovascular disease, previous peripheral vascular disease, previous MI, ejection fraction, disease extent, lesion characteristics (left main disease, bifurcation, long lesion, small diameter), total stent number, total stent length, and clinical diagnosis. To determine a reasonable level of revascularization, the cut-off value of the residual SYNTAX score maximizing the difference of log-rank statistics for POCO was estimated. Additionally, the locally weighted scatterplot smoothing (LOWESS) regression line was used to analyze the association between the estimated risk of POCO and the degree of reduction in anatomical burden, defined by percent change of SYNTAX score [100 × (baseline SYNTAX score-residual SYNTAX score)/baseline SYNTAX score]. All p values were 2-sided, and p < 0.05 was considered statistically significant. The statistical package R, version 3.4.3 (R Foundation for Statistical Computing), was used for statistical analysis.

## Results

### Baseline patient and lesion characteristics

Among a total of 5516 patients, 2003 patients (36.3%) were DM population and 3513 patients (63.7%) were non-DM population (Additional file 1: Fig. S1). Patients and lesion characteristics between the DM and the non-DM populations are described in Additional file 1: Table S2. Compared to the non-DM population, the DM population was older and had higher prevalences of cardiovascular risk factors, including hypertension, dyslipidemia, chronic kidney disease, peripheral vessel disease, prior stroke, and current smoking. The DM population was more likely to be presented as stable angina or silent ischemia than the non-DM population. Regarding the lesion complexity, the DM population had a higher rate of multivessel disease, long lesion, and small vessel disease, resulting in a higher baseline SYNTAX score than the non-DM population. Complete revascularization was achieved in 44.6% in the DM population and 52.2% in the non-DM population (p < 0.001). Patient and lesion characteristics according to the achievement of complete revascularization are described in Table 1. In the DM population, the incomplete revascularization group was more likely to have multivessel disease, long lesions, and small vessel disease than the complete revascularization group. In the non-DM population, the incomplete revascularization group was older and more likely to have chronic kidney disease, previous MI, previous stroke, and complex disease features (multivessel disease, bifurcation lesion, long lesions, and small vessel disease) than the complete revascularization group. The baseline SYNTAX score was higher in the incomplete revascularization group than in the complete revascularization group, regardless of the presence of DM (18.5 ± 8.8 vs.10.8 ± 8.0, p < 0.001 in DM population; 17.4 ± 10.1 vs. 9.5 ± 7.0, p < 0.001 in non-DM population).

**Table 1:** Baseline characteristics

	DM population		p value	Non-DM population		p value
	Complete revascularization (N = 893)	Incomplete revascularization (N = 1110)		Complete revascularization (n = 1833)	Incomplete revascularization (N = 1680)	
Age, years	65.1 ± 9.4	65.2 ± 9.4	0.797	62.3 ± 10.6	65.1 ± 10.4	< 0.001
Male	572 (64.1%)	725 (65.3%)	0.589	1265 (69.0%)	1179 (70.2%)	0.476
Body mass index, kg/m^2^	25.0 ± 3.1	24.9 ± 3.4	0.465	24.7 ± 3.0	24.7 ± 3.0	0.687
Hypertension	654 (73.2%)	848 (76.4%)	0.116	1070 (58.4%)	1051 (62.6%)	0.012
Dyslipidemia	538 (60.2%)	652 (58.7%)	0.524	965 (52.6%)	924 (55.0%)	0.173
Chronic kidney disease	310 (34.7%)	427 (38.5%)	0.092	422 (23.0%)	530 (31.5%)	< 0.001
Peripheral vessel disease	15 (1.7%)	34 (3.0%)	0.065	19 (1.0%)	28 (1.7%)	0.140
Current smoker	181 (20.3%)	278 (25.0%)	0.140	496 (27.1%)	456 (27.1%)	0.986
Prior myocardial infarction	12 (1.3%)	22 (2.0%)	0.355	30 (1.6%)	49 (2.9%)	0.015
Prior stroke	93 (10.4%)	131 (11.8%)	0.364	111 (6.1%)	155 (9.2%)	< 0.001
Family history of CAD	41 (4.6%)	71 (6.4%)	0.099	100 (5.5%)	112 (6.7%)	0.151
LVEF, %	60.9 ± 10.5	59.5 ± 11.1	0.006	62.2 ± 8.9	60.6 ± 10.2	< 0.001
HbA1c, %	7.5 ± 1.6	7.5 ± 1.5	0.449	–	–	–
Presenting with cardiogenic shock	0 (0.0%)	7 (0.6%)	0.046	1 (0.1%)	4 (0.2%)	0.320
Presentations						
Unstable angina	431 (48.3%)	480 (43.2%)	0.118	925 (50.5%)	850 (50.6%)	0.069
Stable angina	398 (44.6%)	530 (47.7%)		820 (44.7%)	719 (42.8%)	
Silent ischemia	64 (7.1%)	100 (9.1%)		88 (4.8%)	111 (6.6%)	
Complexity of disease						
Left main disease	60 (6.7%)	86 (7.7%)	0.427	120 (6.5%)	133 (7.9%)	0.133
Multivessel disease	447 (50.1%)	836 (75.3%)	< 0.001	703 (38.4%)	1133 (67.4%)	< 0.001
At least 1 bifurcation	367 (41.1%)	500 (45.0%)	0.084	648 (35.4%)	774 (46.1%)	< 0.001
At least 1 long lesion	297 (33.3%)	465 (41.9%)	< 0.001	438 (23.9%)	646 (38.5%)	< 0.001
At least 1 small vessel	473 (53.0%)	644 (58.0%)	0.027	684 (37.3%)	841 (50.1%)	< 0.001
Stent number per person	1.7 ± 1.0	1.8 ± 1.0	0.014	1.5 ± 0.8	1.8 ± 1.0	< 0.001
Total stent length, mm	42.0 ± 28.4	45.8 ± 28.1	0.003	35.1 ± 23.4	43.3 ± 27.5	< 0.001
Baseline SYNTAX score	10.8 ± 8.0	18.5 ± 8.8	< 0.001	9.5 ± 7.0	17.4 ± 10.1	< 0.001
Residual SYNTAX score	0.0 ± 0.0	8.5 ± 6.2	< 0.001	0.0 ± 0.0	7.6 ± 6.0	< 0.001
Medication at discharge						
Statin	763 (85.4%)	929 (83.7%)	0.312	1615 (88.1%)	1472 (87.6%)	0.696
ACE-I/ARB	574 (64.3%)	701 (63.2%)	0.636	1016 (55.4%)	981 (58.4%)	0.082
Beta-blocker	467 (52.3%)	654 (58.9%)	0.003	939 (51.2%)	986 (58.7%)	< 0.001
Calcium-channel blocker	295 (33.0%)	336 (30.3%)	0.202	554 (30.2%)	493 (29.3%)	0.595
DM medication at discharge						
Insulin	128 (14.3%)	181 (16.3%)	0.249	–	–	–
Sulfonylurea	245 (27.4%)	311 (28.0%)	0.811	–	–	–
Metformin	298 (33.3%)	372 (33.5%)	0.984	–	–	–
DPP4 inhibitor	116 (13.0%)	135 (12.2%)	0.625	–	–	–
Alpha-glucosidase inhibitor	35 (3.9%)	57 (5.1%)	0.236	–	–	–
TZD	21 (2.3%)	22 (2.0%)	0.680	–	–	–

ACE-I/ARB, angiotensin converting enzyme inhibitor or angiotensin receptor blocker; CAD, coronary artery disease; DM, diabetes mellitus; DPP4, dipeptidyl peptidase 4; HbA1c, glycated hemoglobin; LVEF; left ventricular ejection fraction; SYNTAX, Synergy Between Percutaneous Coronary Intervention With Taxus and Cardiac Surgery; TZD, thiazolidinedione

### Comparisons of patient-oriented and stent-oriented outcomes

**Fig. 1:**
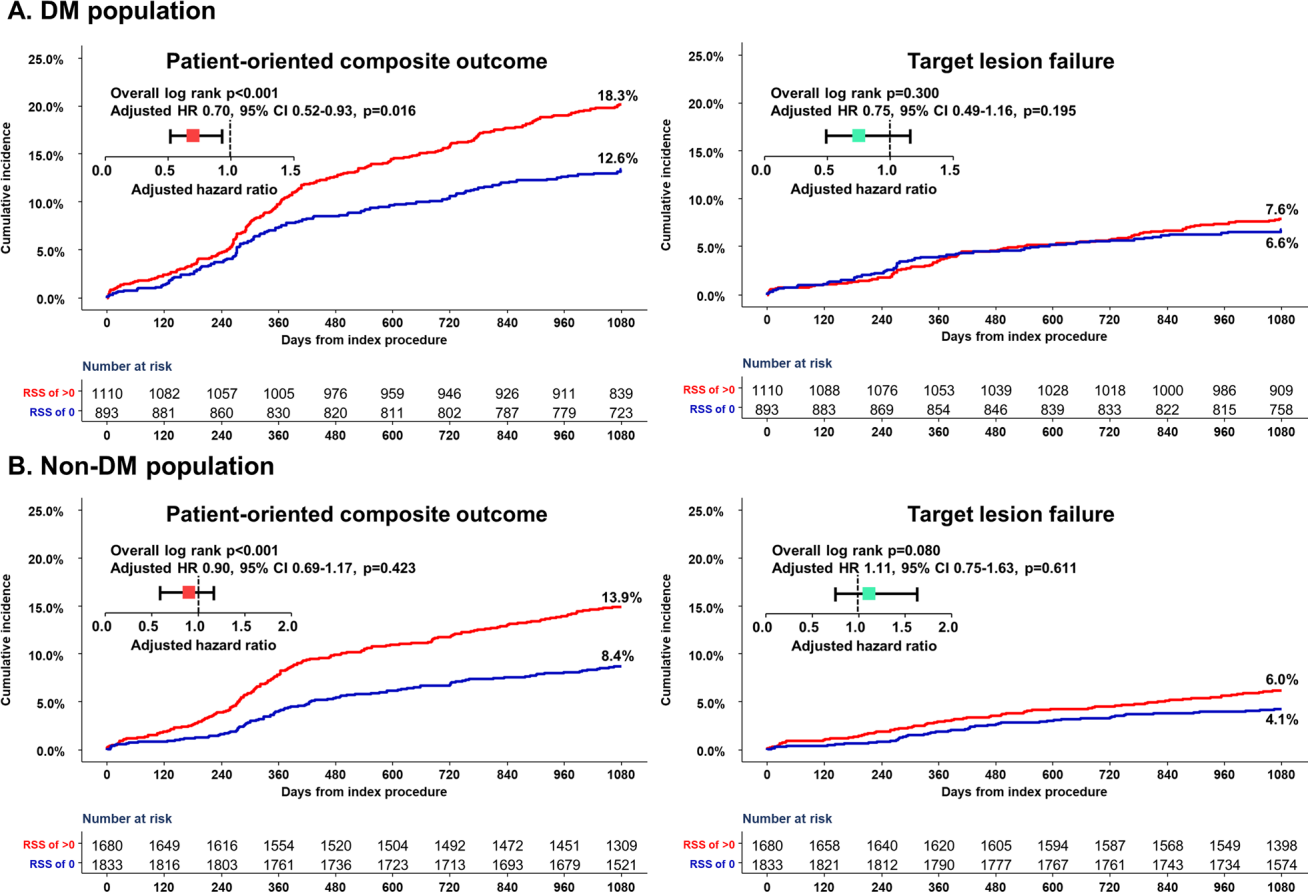
Cumulative incidence of clinical outcomes after complete and incomplete revascularization according to the presence of DM. In the DM population, the complete revascularization group was associated with a lower risk of POCO at three years, but a comparable risk of TLF at three years, compared with the incomplete revascularization group. In the non-DM population, the cumulative incidence of POCO was lower in the complete revascularization group. However, there were no significant differences in the POCO and TLF at three years after covariate adjustment. CI, confidence interval; DM, diabetes mellitus; HR, hazard ratio; POCO, patient-oriented composite outcome; TLF, target lesion failure

The DM population showed a higher risk of POCO at three years than the non-DM population (15.7% vs. 11.0%; adjusted HR 1.31, 95% CI 1.10–1.57, p = 0.003) (Additional file 1: Fig. S2). In the DM population, the complete revascularization group showed a lower cumulative incidence of POCO at three years than the incomplete revascularization group (12.6% vs. 18.3%, p < 0.001), and complete revascularization was associated with a lower risk of POCO at three years (adjusted HR 0.70, 95% CI 0.52–0.93, p = 0.016) (Fig. 1 and Table 2). This result was mainly driven by a lower risk of any revascularization in the complete revascularization group (Table 2). In the non-DM population, the cumulative incidence of POCO was lower in the complete revascularization group than in the incomplete revascularization group (13.9% vs. 8.4%, p < 0.001). However, after adjustment of covariates, no difference was observed in the risk of POCO at three years between the complete and incomplete revascularization groups (adjusted HR 0.90, 95% CI 0.69–1.17, p = 0.423) (Fig. 1 and Table 2).

**Table 2:** Comparison of clinical outcomes between complete and incomplete revascularization according to the presence of DM

DM population	Complete revascularization (N = 893)	Incomplete revascularization (N = 1110)	HR (95% CI)	p value	Adjusted HR^a^ (95% CI)	p value
POCO	112 (12.6%)	202 (18.3%)	0.65 (0.52–0.83)	< 0.001	0.70 (0.52–0.93)	0.016
All-cause death	51 (5.7%)	85 (7.7%)	0.77 (0.54–1.09)	0.142	0.80 (0.51–1.24)	0.322
Any MI	5 (0.6%)	13 (1.2%)	0.56 (0.20–1.61)	0.285	0.94 (0.26–3.33)	0.922
Any revascularization	63 (7.3%)	121 (11.3%)	0.60 (0.44–0.83)	0.002	0.62 (0.42–0.91)	0.016
TLF	58 (6.6%)	83 (7.6%)	0.82 (0.58–1.16)	0.265	0.75 (0.49–1.16)	0.195
Cardiac death	31 (3.5%)	46 (4.2%)	0.81 (0.51–1.30)	0.390	0.69 (0.38–1.26)	0.227
Target-vessel MI	2 (0.2%)	7 (0.7%)	0.48 (0.10–2.35)	0.361	0.45 (0.03–6.27)	0.552
Clinically driven TLR	27 (3.1%)	38 (3.6%)	0.81 (0.49–1.35)	0.414	0.77 (0.41–1.45)	0.418

Non-DM population	Complete revascularization (N = 1833)	Incomplete revascularization (N = 1680)	HR (95% CI)	p value	Adjusted HR^a^ (95% CI)	p value
POCO	153 (8.4%)	233 (13.9%)	0.62 (0.50–0.76)	< 0.001	0.90 (0.69–1.17)	0.423
All-cause death	53 (2.9%)	89 (5.3%)	0.59 (0.42–0.84)	0.003	1.07 (0.68–1.70)	0.757
Any myocardial infarction	7 (0.4%)	14 (0.8%)	0.36 (0.14–0.90)	0.028	0.76 (0.21–2.75)	0.677
Any revascularization	98 (5.4%)	146 (8.8%)	0.63 (0.49–0.81)	< 0.001	0.84 (0.61–1.16)	0.284
TLF	75 (4.1%)	99 (6.0%)	0.76 (0.56–1.03)	0.081	1.11 (0.75–1.63)	0.611
Cardiac death	30 (1.7%)	54 (3.3%)	0.53 (0.34–0.84)	0.006	0.94 (0.51–1.73)	0.847
Target-vessel MI	4 (0.2%)	10 (0.6%)	0.35 (0.11–1.16)	0.085	0.29 (0.04–2.00)	0.213
Clinically driven TLR	44 (2.4%)	43 (2.6%)	1.07 (0.70–1.64)	0.750	1.28 (0.73–2.23)	0.393

^a^The following patient risk factors were included in the multivariate adjusted Cox proportional hazard regression model: age, sex, body mass index, hypertension, hypercholesterolemia, heart failure, chronic kidney disease, family history of CAD, previous cerebrovascular disease, previous peripheral vascular disease, previous MI, ejection fraction, disease extent, lesion characteristics (left main disease, bifurcation, long lesion, small diameter), total stent number, total stent length, and clinical diagnosis

CI, confidence interval; DM, diabetes mellitus; HR, hazard ratio; MI, myocardial infarction; POCO, patient-oriented composite outcome; TLF, target lesion failure; TLR, target lesion revascularization

Even though the DM population showed a higher risk of stent-oriented outcomes than the non-DM population (TLF, 7.2% vs. 5.0%; adjusted HR 1.33, 95% CI 1.02–1.74, p = 0.034) (Figure S2 in Additional file 1), the risks of TLF at three years were not different in the complete and incomplete revascularization groups, regardless of the presence of DM (adjusted HR 0.75, 95% CI 0.49–1.16, p = 0.195 in DM population; adjusted HR 1.11, 95% CI 0.75–1.63, p = 0.611 in non-DM population) (Fig. 1 and Table 2).

### Subgroup analysis in DM population

The clinical benefit of complete revascularization was consistently found in various subgroups regarding POCO at three years, without significant interactions (Additional file 1: Fig. S3 and Table S3). However, the risks of TLF at three years were comparable between the complete and incomplete revascularization groups across the subgroups (Additional file 1: Fig. S3 and Table S4).

### Independent predictors for clinical outcomes according to the presence of DM

In the DM population, the achievement of complete revascularization was independently associated with a lower risk of POCO at three years (adjusted HR 0.68, 95% CI 0.51–0.91, p = 0.009) (Table 3). In addition, multivessel disease, left main disease, and low ejection fraction were independent predictors of POCO in the DM population. In contrast, complete revascularization was not an independent predictor of POCO in the non-DM population. Instead, old age, peripheral vessel disease, and low ejection fraction were independent predictors of POCO in the non-DM population (Table 3).

**Table 3:** Independent predictors of patient-oriented composite outcomes

	Adjusted HR^a^	95% CI	p value
DM population			
Complete revascularization	0.68	0.51–0.91	0.009
Multivessel disease	1.80	1.25–2.59	0.001
LM disease	1.65	1.07–2.56	0.024
LV ejection fraction < 40%	2.14	1.18–3.89	0.012
Non-DM population			
Age, per 1 year increase	1.03	1.01–1.04	< 0.001
Perpheral vessel disease	2.65	1.50–4.66	< 0.001
LV ejection fraction < 40%	2.36	1.18–4.72	0.015

^a^The following patient risk factors were included in the multivariate adjusted Cox proportional hazard regression model: age, sex, body mass index, hypertension, hypercholesterolemia, heart failure, chronic kidney disease, family history of CAD, previous cerebrovascular disease, previous peripheral vascular disease, previous MI, ejection fraction, disease extent, lesion characteristics (left main disease, bifurcation, long lesion, small diameter), total stent number, total stent length, and clinical diagnosis

CI, confidence interval; DM, diabetes mellitus; HR, hazard ratio; LM, left main; LV, left ventricular

### Clinical outcomes according to the level of revascularization in DM population

**Fig. 2:**
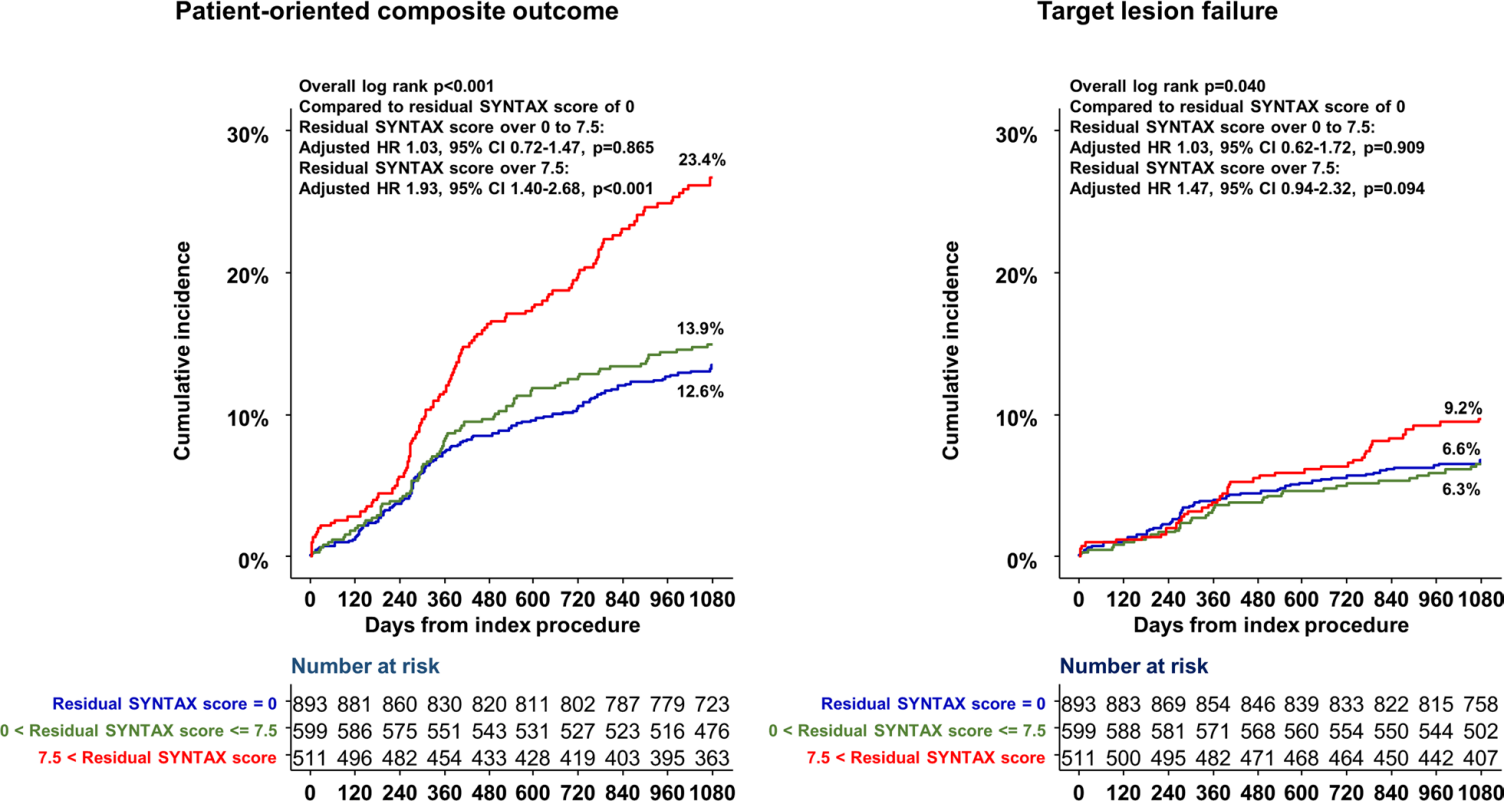
Cumulative incidence of clinical outcomes according to the levels of revascularization in DM population. The cumulative incidence of POCO at three years increased in proportion to the increase of residual SYNTAX score. Patients who had a residual SYNTAX score over a cut-off value of 7.5 was associated with a significantly higher risk of POCO at 3 years compared with those with a residual score of 0. However, this trend was not prominent for TLF at 3 years. CI, confidence interval; DM, diabetes mellitus; HR, hazard ratio; POCO, patient-oriented composite outcome; SYNTAX, Synergy Between Percutaneous Coronary Intervention With Taxus and Cardiac Surgery; TLF, target lesion failure

**Fig. 3:**
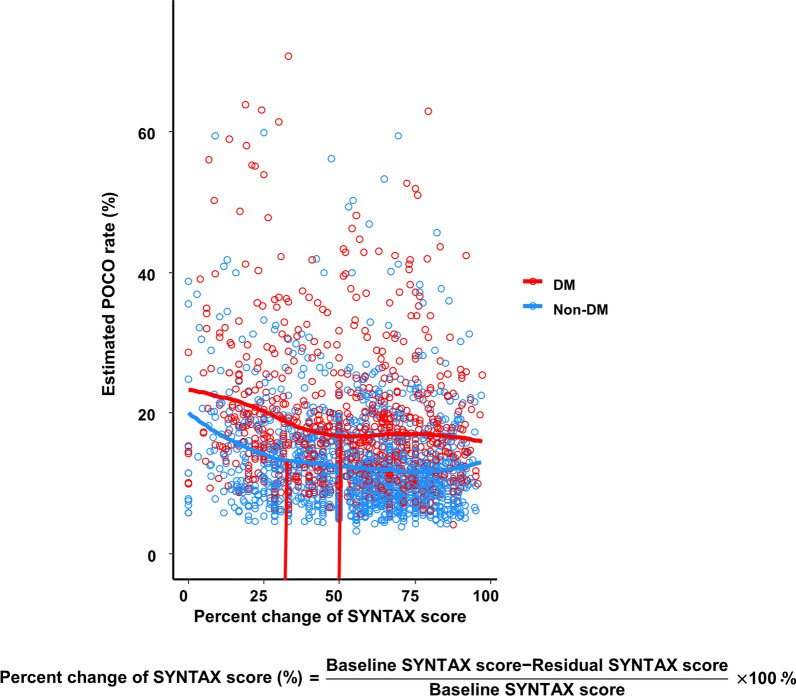
Effect of reduction of anatomical burden on POCO at three years in patients after incomplete revascularization. The association between the degree of anatomical reduction burden and estimated POCO rates at three years are shown according to the presence of DM. The percent change of SYNTAX score was defined as [(baseline SYNTAX score-residual SYNTAX score)/baseline SYNTAX score] × 100%. DM, diabetes mellitus; POCO, patient-oriented composite outcome; SYNTAX, synergy between percutaneous coronary intervention with taxus and cardiac surgery

For the evaluation of a reasonable level of revascularization, the cut-off value of the residual SYNTAX score was calculated in the DM population. The reasonable levels of revascularization were residual SYNTAX score below 7.5 in the DM population (Additional file 1: Fig. S4). When the incomplete revascularization group was further categorized by the cut-off value, the cumulative incidence of POCO was higher in patients with residual SYNTAX score over the cut-off value (definite incomplete revascularization group) than those with residual SYNTAX score under the cut-off value (reasonable incomplete revascularization group) (Fig. 2 and Additional file 1: Table S5). Definite incomplete revascularization group was associated with a higher risk of POCO at 3 years compared with the complete revascularization group (adjusted HR 1.93, 95% CI 1.40–2.68, p < 0.001). However, there was no significant difference in the risk of POCO at 3 years between the reasonable incomplete and complete revascularization groups. The risk of TLF at three years was not significantly changed along with the increase of residual SYNTAX score (Fig. 2 and Additional file 1: Table S5). Additionally, the risk of POCO increased as the percent change of SYNTAX score decreased below 50% in the DM population. However, this trend was prominent with the percent change of SYNTAX score below around 30% in the non-DM population (Fig. 3).

## Discussions

The current study evaluated the clinical impact of angiographic complete revascularization in the DM population who underwent PCI using contemporary DES. The major findings were as follows: First, complete revascularization was associated with a lower risk of POCO at 3 years in the DM population, mainly driven by a lower risk of any revascularization. However, this association was not prominent in the non-DM population. Second, the risks of TLF at three years were not significantly different between the complete and incomplete revascularization groups regardless of the presence of DM. Third, the cumulative incidence of POCO at three years increased along with the increase of residual SYNTAX score in the DM population. However, a significantly higher risk of POCO at three years was only observed in patients with a residual SYNTAX score over 7.5 as compared to patients with complete revascularization. Fourth, the independent predictors of POCO were incomplete revascularization, multivessel disease, left main disease and low ejection fraction in the DM population, and old age, peripheral vessel disease, and low ejection fraction in the non-DM population.

The DM population carries a significantly higher risk of CAD, and its prevalence is still growing globally [23]. It has been demonstrated that CAD in the DM population was more likely to be diffuse disease and multivessel disease [24], and was associated with more significant plaque burden and lipid-rich plaque, prone to rupture [2], which lead to a procedural complexity in reperfusion therapy. DM is also associated with adverse stent-related outcomes after PCI, increasing the risk of stent restenosis or thrombotic obstructions [4–7]. A pooled analysis of BIO-RESOR and BIONYX trials demonstrated that patients with DM had higher risks of target lesion failure than patients without DM after PCI [7]. Furthermore, a recent study by Kim et al. reported a greater plaque progression in the DM population using serial coronary computed tomographic angiography data [25]. All of these findings might lead to worse cardiovascular outcomes and make coronary revascularization more challenging in the DM population [1, 26, 27]. Consistent with the previous studies, the current study showed higher risks of POCO (adjusted HR 1.31, 95% HR 1.10–1.57, p = 0.003) and TLF (adjusted HR 1.33, 95% CI 1.02–1.74, p = 0.034) in the DM than non-DM population. The higher risk of both adverse patient-related and stent-related outcomes raises concerns about whether aggressive revascularization would be beneficial in the DM population. Recent real-world data, including patients with three-vessel CAD, showed that PCI and coronary bypass grafting lowered risks of death and major adverse cardiac and cerebrovascular events compared to medical therapy in CAD patients with DM [28]. Coronary bypass grafting, more complete revascularization, further lowered the risk of major adverse cardiac and cerebrovascular events but did not reduce the risk of death compared to PCI [28]. Although the revascularization strategy in the DM population is still controversial, the current guideline applies the same principles in revascularization strategy irrespective of diabetic status and recommends the placement of new-generation DES [29].

The clinical benefits of complete revascularization in patients with DM have been evaluated in several studies. In a single-center study that included over 500 patients, the DM subgroup with complete revascularization showed a lower risk of cardiac death, myocardial infarction, and revascularization than those with incomplete revascularization [30]. In the post hoc analysis of BARI 2D (Bypass Angioplasty Revascularization Investigation 2 Diabetes) trial, complete revascularization by PCI was associated with a lower risk of a composite of death, myocardial infarction, or stroke, and repeat revascularization compared with incomplete revascularization [31]. However, these studies are limited to apply in the contemporary reperfusion era in that the results were from small study populations, did not specify the stent-related outcomes, and were performed in the 1^st^ generation DES era. The current study evaluated the clinical impact of angiographic complete revascularization in the DM population who underwent PCI using contemporary DES. Among the DM population, the complete revascularization group was associated with a lower risk of POCO at three years but a comparable risk of TLF, compared with the incomplete revascularization group. From this registry-based data, the complexity and extent of coronary disease were more severe in the incomplete revascularization group, and these findings infer potential difficulties in achieving complete revascularization in the incomplete revascularization group. However, the stent-related outcome, TLF, was comparable regardless of the completeness of revascularization and the lesion and procedural complexity in the DM population. Compared with 1st generation drug-eluting stent or bare-metal stent, the superior efficacy and safety of contemporary stents may explain these results [32, 33]. The difference shown for the risk of POCO was mainly contributed by a higher risk of any revascularization in the incomplete revascularization group. These results would suggest the clinical importance of targeting complete revascularization in the DM population as the progression of residual disease in the non-stented segment remained after incomplete revascularization may result in an increased burden of subsequent revascularization.

Interestingly, a significantly higher risk of POCO at three years was only observed in patients with a residual SYNTAX score of over 7.5 but not in those with a residual score below 7.5 compared with the complete revascularization group. Additionally, in the DM population, the risk of POCO at three years decreased as the percent change of the SYNTAX score (the parameter which reflects the extent of reducing residual disease) increased up to 50%, which, however, remained similar beyond 50%. These results would implicate an existence of a reasonable degree of revascularization where the additional benefits could not be expected by more aggressive revascularization. In contrast, no significant differences were observed regarding the patient- and stent-oriented outcomes between the complete and incomplete revascularization groups in the non-DM population. Additionally, we observed that the risk of POCO was similar when the percent change of the SYNTAX score increased more than around 30% in the non-DM population. In sum, these results suggest that relieving residual disease would be more critical in the DM population than the non-DM population.

The current study results are consistent with the findings from previous studies demonstrating the clinical benefit of complete revascularization in CAD patients with DM [30, 31]. However, our results further provided the profile of clinical events after PCI with 2nd generation DES in that the difference of the clinical events between complete and incomplete revascularization groups was caused by the residual disease in the non-stented segments in patients with DM. Furthermore, not all residual diseases after PCI were associated with worse clinical outcomes, and the disease burden of residual disease was important for deciding the patients’ prognosis. However, coronary stenting at all visible lesions is not always reasonable and sometimes not possible. Our results also suggest that there might be a reasonable level of revascularization in patients with DM. Considering the nature of CAD in DM, not only the residual disease burden but also plaque vulnerability or physiologic status can also be associated with the clinical outcomes [2, 24, 34]. Recently, the detailed characteristics of geometry and vulnerable features of plaques in patients with DM have been reported in various studies with advanced intracoronary imaging techniques and physiologic parameters [11, 34, 35]. Therefore, a comprehensive approach combining various modalities based on the understanding of the nature of DM in CAD would lead to an optimal level of revascularization in the DM population by defining the clinical significance of residual disease after PCI.

## Study limitations

There are several limitations to be concerned. First, this study was from an observational registry, not from a randomized controlled study. The achievement of complete revascularization depended upon the complexity of the disease and the physician’s discretion. Therefore, the study results have to be considered as hypothesis-generating. Second, the complexity and extent of coronary disease were more severe in the incomplete revascularization group than in the complete revascularization group. Although we tried to adjust these differences between the two groups, these results suggest potential difficulties in achieving complete revascularization in the incomplete revascularization group. In these regards, further prospective studies are warranted. Third, the current study focused on the residual anatomical disease burden after PCI and did not evaluate the hemodynamic significance of the lesions or lesion vulnerability by intracoronary imaging. Therefore, the effects of these factors cannot be explained in this study. Fourth, the data for the timing of revascularization was lacking. However, most of the patients underwent elective PCI in our study, except for the small number of patients presenting with cardiogenic shock (0.2%). Finally, strict glucose level control was not achieved in the DM population in our study. Therefore, there could be a potential confounding effect related to the glucose level affecting the clinical outcomes. However, we observed a significant trend for the improvement in HbA1c (%) level during follow-up after PCI in the DM population (p for trend < 0.001) (Additional file 1: Fig. S5). Furthermore, this trend was consistent in both complete (p for trend 0.002) and incomplete revascularization (p for trend < 0.001) groups (Additional file 1: Fig. S5). No significant differences were observed in HbA1c (%) levels between the two groups at baseline and annual follow-up periods.

## Conclusions

The clinical benefit of angiographic complete revascularization is more prominent in patients with DM than without DM after three years of follow-up. Relieving residual disease might be more critical in the DM population than the non-DM population.

## Supplementary Information

**Additional file 1: Table S1.** List of investigators and participating centers of the Grand Drug-Eluting Stent registry. **Table S2.** Comparison of baseline characteristics between DM and non-DM population. **Table S3.** Subgroup analysis for the risk of patient-oriented composite outcome after complete revascularization compared to incomplete revascularization in the DM population. **Table S4.** Subgroup analysis for the risk of target lesion failure after complete revascularization compared to incomplete revascularization in the DM population. **Table S5.** Clinical outcomes according to the residual SYNTAX score in DM population. **Figure S1.** Study flow. **Figure S2.** Comparisons of clinical outcomes between DM and non-DM populations. **Figure S3.** Subgroup analysis for the risk of clinical outcomes after complete revascularization compared to incomplete revascularization in DM population. **Figure S4.** Reasonable level of revascularization in DM population. **Figure S5.** Annual trends of HbA1c (%) level among DM population.

## Data Availability

Due to ethical restrictions by the ethics committees of each participating center, the data underlying this study cannot be made publicly available, as public availability would compromise patient confidentiality and participant privacy. Therefore, access to aggregated data will be granted after review by the GRAND-DES steering committee; the access requests can be sent to hyosoo@snu.ac.kr.

## Abbreviations

CAD: Coronary artery disease
DES: Drug-eluting stent
DM: Diabetes mellitus
MI: Myocardial infarction
PCI: Percutaneous coronary intervention
POCO: Patient-oriented composite outcome
SYNTAX score: SYNergy between percutaneous coronary intervention with TAXus and cardiac surgery score
TLF: Target lesion failure
