# Supplementary material for: Angiographic complete revascularization versus incomplete revascularization in patients with diabetes mellitus
Source: Cardiovasc Diabetol. 2022 Apr 19;21:56. doi: 10.1186/s12933-022-01488-7 (PMC9019985; doi:10.1186/s12933-022-01488-7)

**Additional file 1: Materials**

**Angiographic Complete Revascularization Versus Incomplete Revascularization in patients with Diabetes Mellitus**

Doyeon Hwang, MD^✝^, Jiesuck Park, MD^✝^, Han-Mo Yang, MD, PhD, Seokhun Yang, MD, Jeehoon Kang, MD, Jung-Kyu Han, MD, PhD, Kyung Woo Park, MD, PhD, MBA, Hyun-Jae Kang, MD, PhD, Bon-Kwon Koo, MD, PhD, Hyo-Soo Kim, MD, PhD

**Table S1. List of investigators and participating centers of the Grand Drug-Eluting Stent registry**

| **No.** | **Name** | **Affiliation** |
| --- | --- | --- |
| 1 | Sang-Wook Lim | CHA Bundang Medical Center |
| 2 | Jei Keon Chae | Chonbuk National University Hospital |
| 3 | Myung Ho Jeong | Chonnam National University Hospital |
| 4 | Jang-Whan Bae | Chungbuk National University Hospital |
| 5 | In-Whan Seong / Jin-Ok Jeong | Chungnam National University Hospital |
| 6 | Kee-Sik Kim | Daegu Catholic University Medical Center |
| 7 | Bong-Ryeol Lee | Daegu Fatima Hospital |
| 8 | Tae Soo Kang | Dankook University Hospital |
| 9 | Young-Dae Kim | Dong-A University Hospital |
| 10 | Si Hoon Park | Ewha Womans University Mokdong Hospital |
| 11 | Tae Hoon Ahn | Gacheon University Gil Hospital |
| 12 | Hyuck Moon Kwon | Gangnam Severance Hospital |
| 13 | Wan Kim | Gwangju Veterans Hospital |
| 14 | Yong Whi Park / Jin-Yong Hwang / Choong Hwan Kwak | Gyeongsang National University Hospital |
| 15 | Kyoo-Rok Han | Hallym University Kangdong Sacred Heart Hospital |
| 16 | Young Jin Choi | Hallym University Sacred Heart Hospital |
| 17 | Kyung-Soo Kim | Hanyang University Medical Center |
| 18 | Gi Chang Kim | Incheon Sarang Hospital |
| 19 | Keum-Soo Park / Seong-Ill Woo | Inha University Hospital |
| 20 | Dae-Kyung Kim / Jae-Sik Jang | Inje University Busan Paik Hospital |
| 21 | Doo-Il Kim | Inje University Haeundae Paik Hospital |
| 22 | Seong Yun Lee | Inje University Ilsan Paik Hospital |
| 23 | Song-Yi Kim | Jeju National University Hospital |
| 24 | Jin-Ho Kang / Bum-Soo Kim | Kangbuk Samsung Hospital |
| 25 | Seung-Ho Hur | Keimyung University Dongsan Medical Center |
| 26 | Jang-Ho Bae | Konyang University Hospital |
| 27 | Do-Sun Lim | Korea University Anam Hospital |
| 28 | Seung-Woon Rha | Korea University Guro Hospital |
| 29 | Tae-Joon Cha | Kosin University Gospel Hospital |
| 30 | Seung-Uk Lee | Kwangju Christian Hospital |
| 31 | Jin-Man Cho | Kyunghee University Gangdong Hospital |
| 32 | Myeong-Gon Kim | Kyunghee University Hospital |
| 33 | Hun-Sik Park | Kyungpook National University Hospital |
| 34 | Dong Woon Jeon | National Health Insurance Corporation Ilsan Hospital |
| 35 | Jay Young Rhew | Presbyterian Medical Center |
| 36 | Han Cheol Lee | Pusan National University Hospital |
| 37 | Kook-Jin Chun | Pusan National University Yangsan Hospital |
| 38 | Hyeon-Cheol Kwon | Samsung Medical Center |
| 39 | Jin-Sik Park / Rak-Kyeong Choi | Sejong General Hospital |
| 40 | Sang-Hyun Kim / Jae-Bin Seo | Seoul National University Boramae Medical Center |
| 41 | Dong-Ju Choi / In-Ho Chae | Seoul National University Bundang Hospital |
| 42 | Hyo-Soo Kim | Seoul National University Hospital |
| 43 | Dong-Kyu Jin | Soonchunhyang University Cheonan Hospital |
| 44 | Do Hoi Kim | Soonchunhyang University Gumi Hospital |
| 45 | Min-Su Hyon | Soonchunhyang University Seoul Hospital |
| 46 | Jang Hyun Cho | St. Carollo Hospital |
| 47 | Sung Ho Her | The Catholic University of Korea, Daejeon St. Mary's Hospital |
| 48 | Ki-Bae Seung | The Catholic University of Korea, Seoul St. Mary's Hospital |
| 49 | Geon-Woong Moon | The Catholic University of Korea, St. Vincent Hospital |
| 50 | Hui-Kyung Jeon | The Catholic University of Korea, Uijeongbu St. Mary's Hospital |
| 51 | Eun-Seok Shin | Ulsan University Hospital |
| 52 | Jung Han Yoon | Wonju Severance Christian Hospital |
| 53 | Seok-Kyu Oh | Wonkwang University Hospital |
| 54 | Jong-Seon Park | Yeungnam University Hospital |
| 55 | Yang-Soo Jang | Yonsei University Severance Hospital |

**Table S2. Comparison of baseline characteristics between DM and non-DM population**

|  | **DM population**  **(N=2,003)** | **Non-DM population**  **(N=3,513)** |  |
| --- | --- | --- | --- |
| Age, years | 65.2±9.4 | 63.7±10.6 | <0.001 |
| Male | 1,297 (64.8%) | 2,444 (69.6%) | <0.001 |
| Body mass index, kg/m^2^ | 25.0±3.3 | 24.7±3.0 | 0.004 |
| Hypertension | 1,502 (75.0%) | 2,123 (60.4%) | <0.001 |
| Dyslipidemia | 1,190 (59.4%) | 1,889 (53.8%) | <0.001 |
| Chronic kidney disease | 737 (41.4%) | 952 (30.4%) | <0.001 |
| Peripheral vessel disease | 49 (2.5%) | 47 (1.3%) | 0.002 |
| Current smoker | 459 (22.9%) | 952 (27.1%) | 0.001 |
| Prior myocardial infarction | 34 (1.7%) | 79 (2.2%) | 0.197 |
| Prior stroke | 224 (11.2%) | 266 (7.6%) | <0.001 |
| Family history of CAD | 112 (5.6%) | 212 (6.0%) | 0.540 |
| LVEF, % | 60.1±10.9 | 61.4±9.5 | <0.001 |
| HbA1c, % | 7.5±1.5 | – | – |
| Presenting with cardiogenic shock | 7 (0.3%) | 5 (0.1%) | 0.198 |
| Presentations |  |  |  |
| Unstable angina | 911 (45.5%) | 1,775 (50.6%) | <0.001 |
| Stable angina | 928 (46.3%) | 1,539 (43.8%) |  |
| Silent ischemia | 164 (8.2%) | 199 (5.6%) |  |
| Complexity of disease |  |  |  |
| Left main disease | 146 (7.3%) | 253 (7.2%) | 0.947 |
| Multivessel disease | 1,283 (64.1%) | 1,836 (52.3%) | <0.001 |
| At least 1 bifurcation | 867 (43.3%) | 1,422 (40.5%) | 0.043 |
| At least 1 long lesion | 762 (38.0%) | 1,084 (30.8%) | <0.001 |
| At least 1 small vessel | 1,117 (55.8%) | 1,525 (43.4%) | <0.001 |
| Stent number per person | 1.8±1.0 | 1.6±0.9 | <0.001 |
| Total stent length, mm | 44.1±28.3 | 39.0±25.8 | <0.001 |
| Baseline SYNTAX score | 15.0±9.3 | 13.3±9.5 | <0.001 |
| Residual SYNTAX score | 4.7±6.3 | 3.6±5.6 | <0.001 |
| Complete revascularization | 893 (44.6%) | 1,834 (52.2%) | <0.001 |
| Medication at discharge |  |  |  |
| Statin | 1,692 (84.5%) | 3,087 (87.9%) | <0.001 |
| ACE-I/ARB | 1,275 (63.7%) | 1,997 (56.8%) | <0.001 |
| Beta-blocker | 1,121 (55.9%) | 1,925 (54.8%) | 0.417 |
| Calcium-channel blocker | 631 (31.5%) | 1,047 (29.8%) | 0.198 |
| Insulin | 309 (15.4%) | – | – |
| Sulfonylurea | 556 (27.7%) | – | – |
| Metformin | 670 (33.4%) | – | – |
| DPP4 inhibitor | 251 (12.5%) | – | – |
| a-glucosidase inhibitor | 92 (4.6%) | – | – |
| TZD | 43 (2.1%) | – | – |

Abbreviations: ACE-I/ARB, angiotensin converting enzyme inhibitor or angiotensin receptor blocker; CAD, coronary artery disease; DM, diabetes mellitus; DPP4, dipeptidyl peptidase 4; HbA1c, glycated hemoglobin; LVEF; left ventricular ejection fraction; SYNTAX, Synergy Between Percutaneous Coronary Intervention With Taxus and Cardiac Surgery; TZD, thiazolidinedione.

**Table S3. Subgroup analysis for the risk of patient-oriented composite outcome after complete revascularization compared to incomplete revascularization in the DM population**

|  | **Patient number** | **Adjusted HR^*^** | **95% CI** | **p value** | **Interaction p value** |
| --- | --- | --- | --- | --- | --- |
| ***Clinical characteristics*** | | | | | |
| **Age≥70 years** | 725 | 0.61 | 0.38-0.97 | 0.035 | 0.962 |
| **Age<70 years** | 1,278 | 0.69 | 0.46-1.03 | 0.069 |  |
| **Male** | 1,297 | 0.65 | 0.45-0.95 | 0.024 | 0.709 |
| **Female** | 706 | 0.84 | 0.50-1.41 | 0.501 |  |
| **With Dyslipidemia** | 1,190 | 0.82 | 0.56-1.21 | 0.317 | 0.315 |
| **Without Dyslipidemia** | 813 | 0.56 | 0.35-0.91 | 0.018 |  |
| **EF<40%** | 41 | 0.43 | 0.10-1.84 | 0.254 | 0.543 |
| **EF≥40%** | 1,707 | 0.73 | 0.53-0.99 | 0.048 |  |
| **With CKD** | 737 | 0.77 | 0.51-1.16 | 0.211 | 0.149 |
| **Without CKD** | 1,043 | 0.59 | 0.38-0.92 | 0.020 |  |
| ***Lesion characteristics*** | | | | | |
| **Multivessel disease** | 1,283 | 0.65 | 0.46-0.92 | 0.014 | 0.779 |
| **Single vessel disease** | 720 | 0.59 | 0.28-1.22 | 0.154 |  |
| **LM disease** | 146 | 0.27 | 0.50-1.42 | 0.121 | 0.386 |
| **Without LM disease** | 1,857 | 0.71 | 0.52-0.97 | 0.032 |  |
| **Bifurcation lesion** | 867 | 0.79 | 0.51-1.20 | 0.266 | 0.608 |
| **Without bifurcation lesion** | 1,136 | 0.65 | 0.42-1.01 | 0.053 |  |
| **Long lesion** | 762 | 0.74 | 0.47-1.16 | 0.191 | 0.716 |
| **Without long lesion** | 1,240 | 0.66 | 0.44-0.99 | 0.044 |  |

*The following patient risk factors were included in the multivariate adjusted Cox proportional hazard regression model: age, sex, body mass index, hypertension, hypercholesterolemia, heart failure, chronic kidney disease, family history of CAD, previous cerebrovascular disease, previous peripheral vascular disease, previous MI, ejection fraction, disease extent, lesion characteristics (left main disease, bifurcation, long lesion, small diameter), total stent number, total stent length, and clinical diagnosis.

Abbreviations: CAD, coronary artery disease; CI, confidence interval; CKD, chronic kidney disease; DM, diabetes mellitus; EF, ejection fraction; HR, hazard ratio; HTN, hypertension; LM, left main.

**Table S4. Subgroup analysis for the risk of target lesion failure after complete revascularization compared to incomplete revascularization in the DM population**

|  | **Patient number** | **Adjusted HR^*^** | **95% CI** | **p value** | **Interaction p value** |
| --- | --- | --- | --- | --- | --- |
| ***Clinical characteristics*** | | | | | |
| **Age≥70 years** | 725 | 0.57 | 0.29-1.12 | 0.103 | 0.425 |
| **Age<70 years** | 1,278 | 0.88 | 0.47-1.64 | 0.687 |  |
| **Male** | 1,297 | 0.74 | 0.44-1.25 | 0.256 | 0.726 |
| **Female** | 706 | 0.73 | 0.31-1.74 | 0.481 |  |
| **With Dyslipidemia** | 1,190 | 0.98 | 0.55-1.75 | 0.946 | 0.514 |
| **Without Dyslipidemia** | 813 | 0.56 | 0.28-1.13 | 0.105 |  |
| **EF<40%** | 41 | 0.47 | 0.09-2.36 | 0.361 | 0.247 |
| **EF≥40%** | 1,707 | 0.84 | 0.53-1.33 | 0.453 |  |
| **With CKD** | 737 | 0.98 | 0.55-1.75 | 0.946 | 0.231 |
| **Without CKD** | 1,043 | 0.54 | 0.27-1.09 | 0.084 |  |
| ***Lesion characteristics*** | | | | | |
| **Multivessel disease** | 1,283 | 0.56 | 0.29-1.06 | 0.075 | 0.254 |
| **Single vessel disease** | 720 | 1.04 | 0.33-3.25 | 0.944 |  |
| **LM disease** | 146 | 0.18 | 0.02-1.45 | 0.107 | 0.145 |
| **Without LM disease** | 1,857 | 0.88 | 0.55-1.39 | 0.576 |  |
| **Bifurcation lesion** | 762 | 0.65 | 0.35-1.23 | 0.186 | 0.565 |
| **Without bifurcation lesion** | 1,240 | 0.86 | 0.46-1.63 | 0.645 |  |
| **Long lesion** | 1,283 | 0.77 | 0.38-1.55 | 0.461 | 0.706 |
| **Without long lesion** | 720 | 0.84 | 0.46-1.54 | 0.569 |  |

*The following patient risk factors were included in the multivariate adjusted Cox proportional hazard regression model: age, sex, body mass index, hypertension, hypercholesterolemia, heart failure, chronic kidney disease, family history of CAD, previous cerebrovascular disease, previous peripheral vascular disease, previous MI, ejection fraction, disease extent, lesion characteristics (left main disease, bifurcation, long lesion, small diameter), total stent number, total stent length, and clinical diagnosis.

Abbreviations: CAD, coronary artery disease; CI, confidence interval; CKD, chronic kidney disease; DM, diabetes mellitus; EF, ejection fraction; HR, hazard ratio; HTN, hypertension; LM, left main.

**Table S5. Clinical outcomes according to the residual SYNTAX score in DM population**

|  | **Events (%)** | **HR (95% CI)** | **Adjusted HR^*^**  **(95% CI)** | **HR (95% CI)** | **Adjusted HR^*^**  **(95% CI)** |
| --- | --- | --- | --- | --- | --- |
| **POCO** |  |  |  |  |  |
| Residual SYNTAX Score=0 (N=893) | 112 (12.6%) | Reference | Reference |  |  |
| Residual SYNTAX Score >0 to 7.5 (N=599) | 83 (13.9%) | 1.16 (0.87-1.55) | 1.03 (0.72-1.47) | Reference | Reference |
| Residual SYNTAX Score >7.5 (N=511) | 118 (23.4%) | 2.00 (1.53-2.60) | 1.93 (1.40-2.68) | 1.72 (1.30-2.27) | 1.87 (1.34-2.62) |
| **TLF** |  |  |  |  |  |
| Residual SYNTAX Score=0 (N=893) | 58 (6.6%) | Reference | Reference |  |  |
| Residual SYNTAX Score >0 to 7.5 (N=599) | 37 (6.3%) | 1.02 (0.68-1.55) | 1.03 (0.62-1.72) | Reference | Reference |
| Residual SYNTAX Score >7.5 (N=511) | 46 (9.2%) | 1.46 (0.98-2.17) | 1.47 (0.94-2.32) | 1.42 (0.92-2.19) | 1.65 (0.58-1.62) |

*The following patient risk factors were included in the multivariate adjusted Cox proportional hazard regression model: age, sex, body mass index, hypertension, hypercholesterolemia, heart failure, chronic kidney disease, family history of CAD, previous cerebrovascular disease, previous peripheral vascular disease, previous MI, ejection fraction, disease extent, lesion characteristics (left main disease, bifurcation, long lesion, small diameter), total stent number, total stent length, and clinical diagnosis.

Abbreviations: CI, confidence interval; DM, diabetes mellitus; HR, hazard ratio; POCO, patient-oriented composite outcome; TLF, target lesion failure; SYNTAX, Synergy Between Percutaneous Coronary Intervention With Taxus and Cardiac Surgery.

**Figure Legends**

**Figure S1. Study flow**

From the Grand-DES registry, we analyzed 5,516 patients who underwent PCI using 2^nd^ generation DES and were available with SYNTAX score. Patients with initial presentation of acute myocardial infarction or those with a previous history of coronary revascularization were excluded.

Abbreviations: AMI, acute myocardial infarction; DES, drug-eluting stent; DM, diabetes mellitus; SYNTAX, Synergy Between Percutaneous Coronary Intervention With Taxus and Cardiac Surgery.

**Figure S2. Comparisons of clinical outcomes between DM and non-DM populations**

The DM population showed higher risks of patient-oriented composite outcome and target lesion failure at 3 years than the non-DM population.

Abbreviations: CI, confidence interval; DM, diabetes mellitus; HR, hazard ratio.

**Figure S3. Subgroup analysis for the risk of clinical outcomes after complete revascularization compared to incomplete revascularization in DM population**

Consistent trends were found across the various subgroups regarding the clinical benefit of complete revascularization for the risk of POCO at 3 years.

Abbreviations: CKD, chronic kidney disease; EF, ejection fraction; HTN, hypertension; LM, left-main coronary artery; POCO, patient-oriented composite outcome.

**Figure S4. Reasonable level of revascularization in DM population**

To determine the reasonable level of revascularization, cut-off values of the residual SYNTAX score were calculated based on maximizing the difference of log-rank statistics for clinical outcomes.

Abbreviations: DM, diabetes mellitus; SYNTAX, Synergy Between Percutaneous Coronary Intervention With Taxus and Cardiac Surgery.

**Figure S5. Annual trends of HbA1c (%) level among DM population**

Annual HbA1c (%) levels in the DM population are shown. Overall, the HbA1c (%) level showed a decreasing trend during follow-up (p for trend <0.001). This trend was consistent in both complete (p for trend 0.002) and incomplete revascularization (p for trend <0.001) groups. No significant differences were observed in HbA1c (%) levels between the two groups at baseline and annual follow-up periods.

Abbreviations: DM, diabetes mellitus


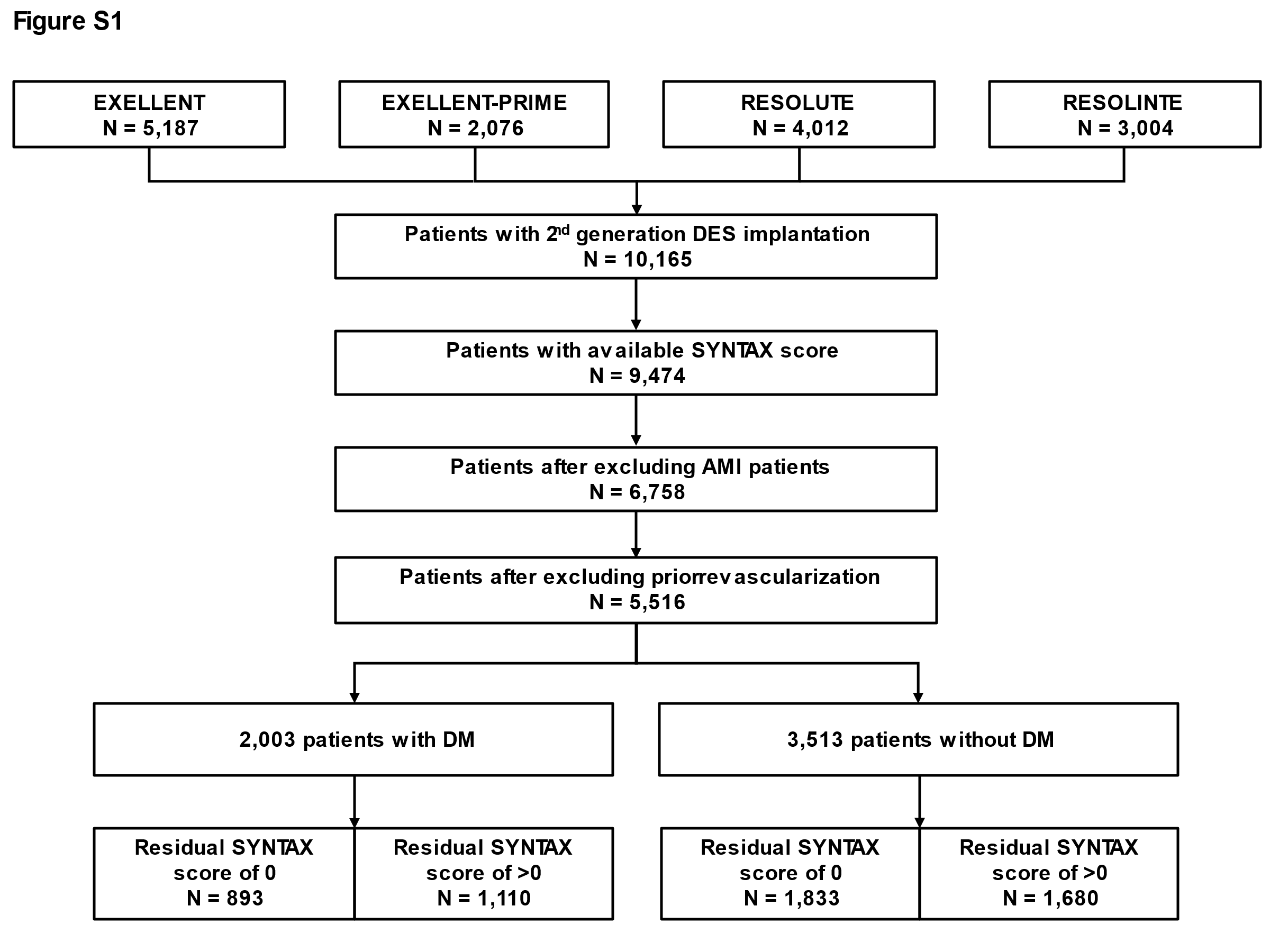

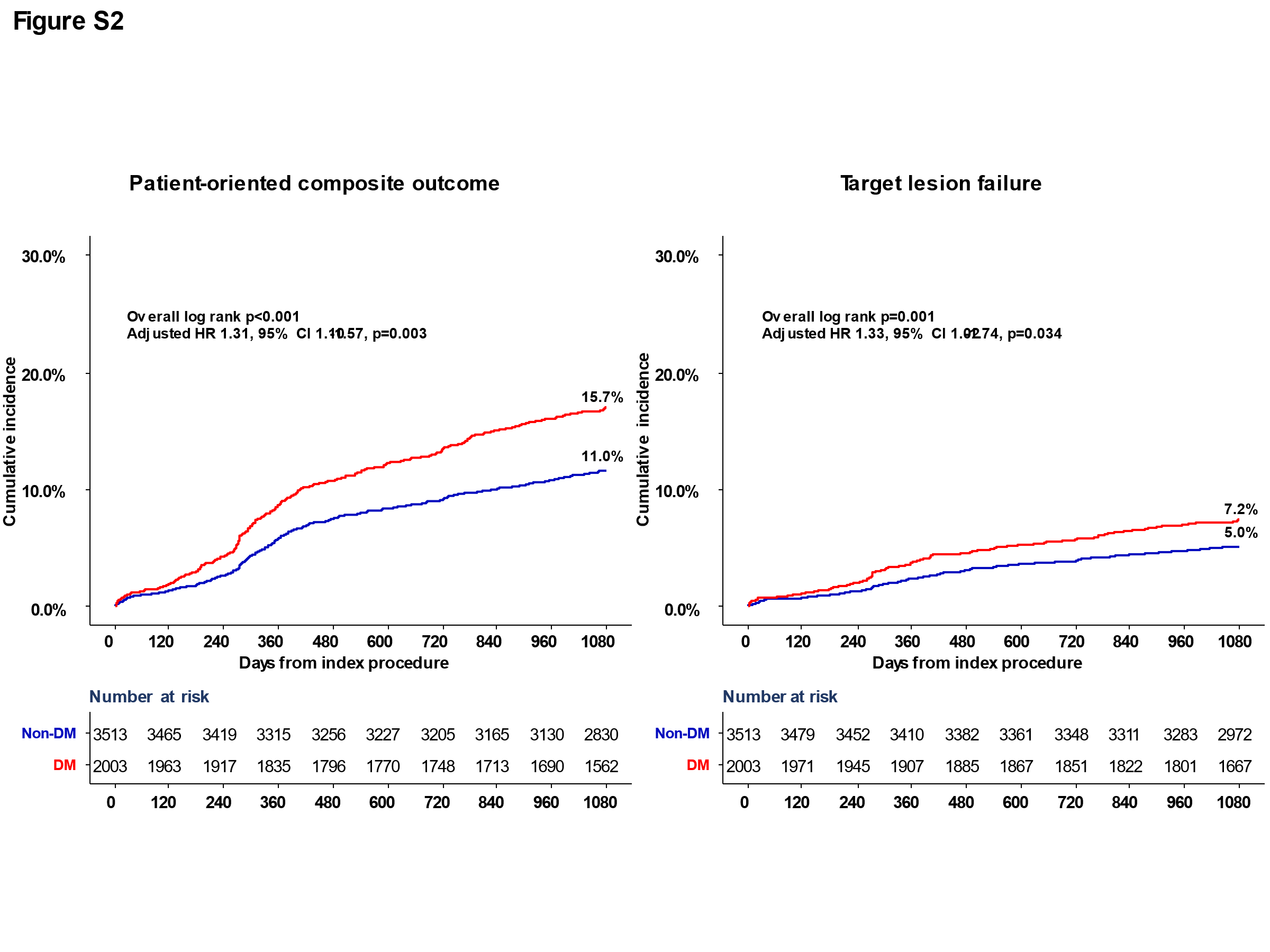

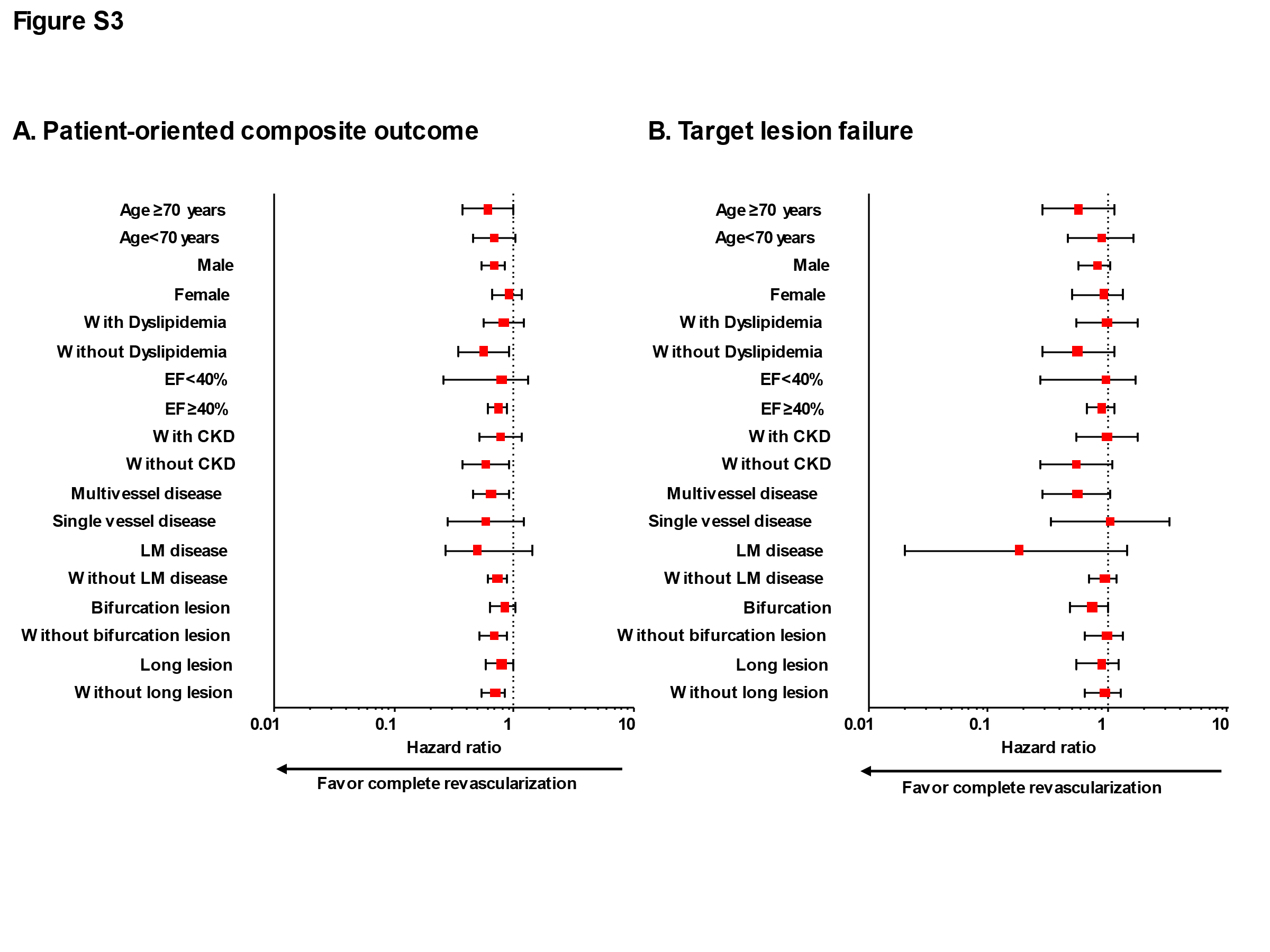

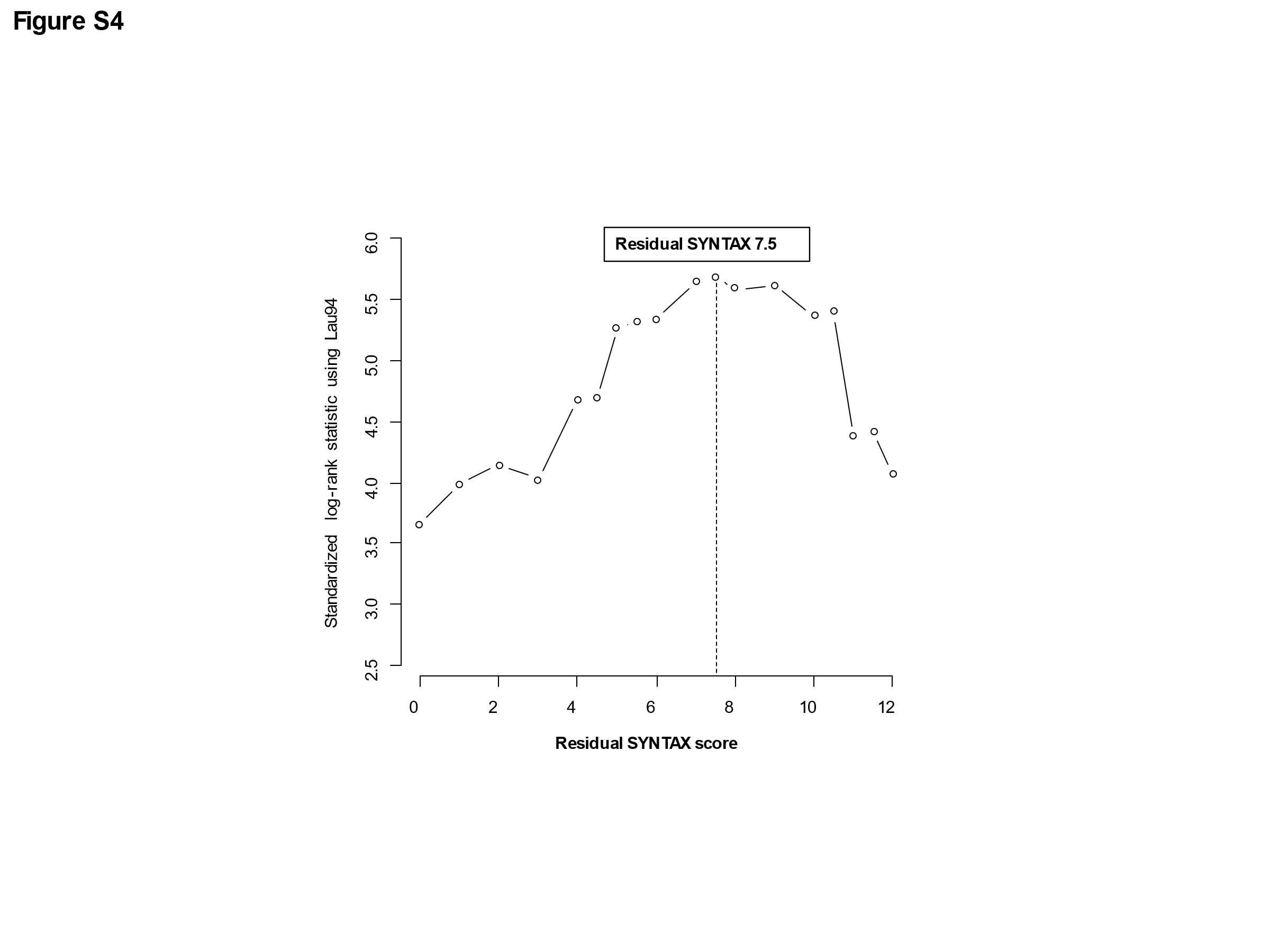

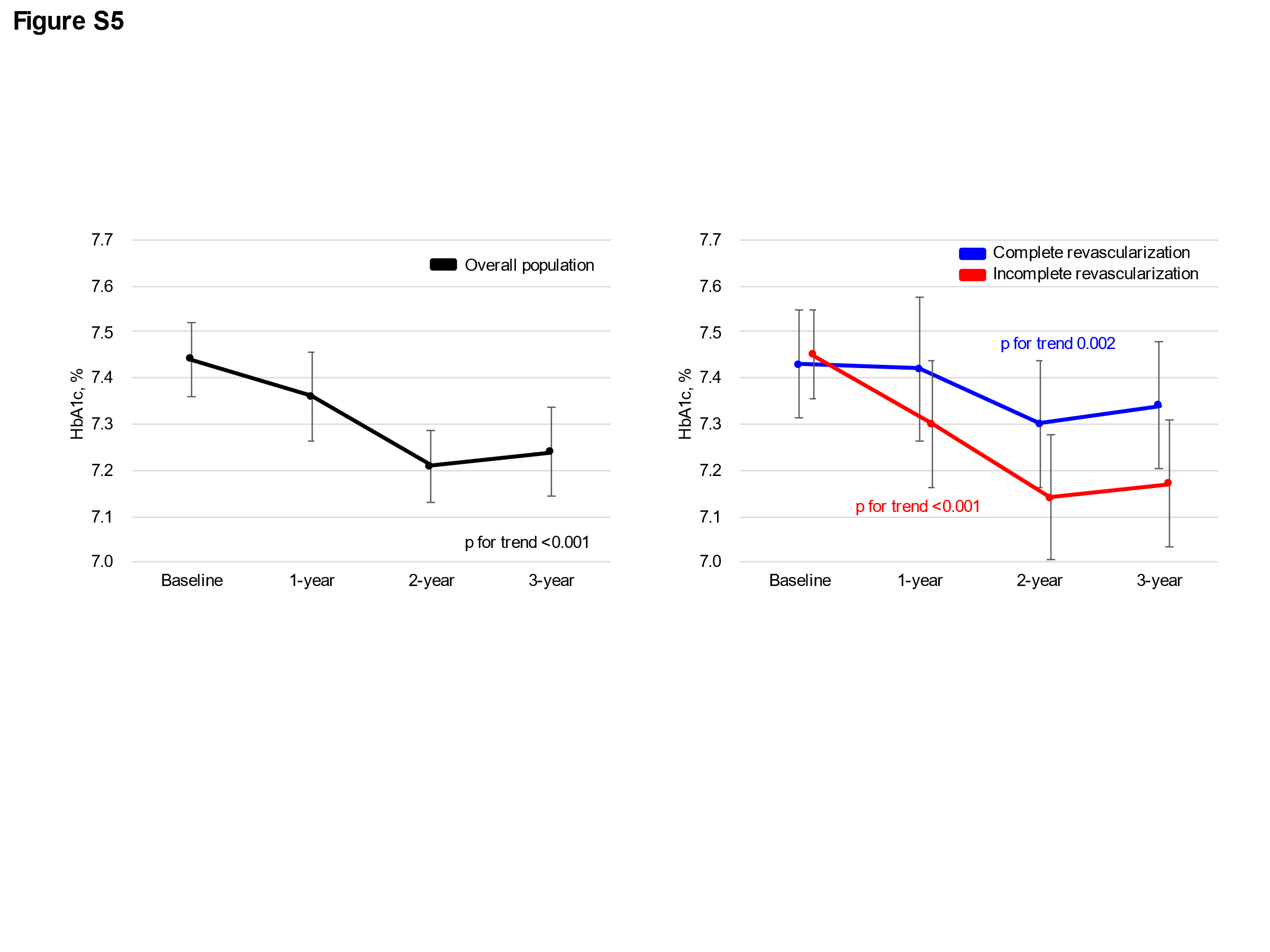

Supplement: Supplementary file 1 — Additional file 1: Table S1. List of investigators and participating centers of the Grand Drug-Eluting Stent registry. Table S2. Comparison of baseline characteristics between DM and non-DM population. Table S3. Subgroup analysis for the risk of patient-oriented composite outcome after complete revascularization compared to incomplete revascularization in the DM population. Table S4. Subgroup analysis for the risk of target lesion failure after complete revascularization compared to incomplete revascularization in the DM population. Table S5. Clinical outcomes according to the residual SYNTAX score in DM population. Figure S1. Study flow. Figure S2. Comparisons of clinical outcomes between DM and non-DM populations. Figure S3. Subgroup analysis for the risk of clinical outcomes after complete revascularization compared to incomplete revascularization in DM population. Figure S4. Reasonable level of revascularization in DM population. Figure S5. Annual trends of HbA1c (%) level among DM population. [file 12933_2022_1488_MOESM1_ESM.docx]
